# Association between urinary caffeine and caffeine metabolites and stroke in American adults: a cross-sectional study from the NHANES, 2009–2014

**DOI:** 10.1038/s41598-023-39126-1

**Published:** 2023-07-22

**Authors:** Jinming Fan, Yajun Yuan, Xiaoting Zhang, Wenhan Li, Wuqin Ma, Wenhao Wang, Jinyan Gu, Bin Zhou

**Affiliations:** 1 Center of Cerebrovascular Disease, The Fifth Affiliated Hospital of Sun Yat-Sen University, Zhuhai, 519000 Guangdong Province China; 2 Center of Interventional Medicine, The Fifth Affiliated Hospital of Sun Yat-Sen University, Zhuhai, 519000 Guangdong Province China; 3 Department of Scientific Research, The Fifth Affiliated Hospital of Sun Yat-Sen University, Zhuhai, 519000 Guangdong Province China

**Keywords:** Biomarkers, Diseases, Neurology, Risk factors

## Abstract

This study investigates the potential correlation between urinary caffeine levels and the occurrence of stroke, a serious cerebrovascular disease that can lead to disability or death. The data used in this study was obtained from the National Health and Nutrition Examination Survey conducted between 2009 and 2014. The study analyzed a total of 5,339 individuals, divided into a control group (n = 5,135) and a stroke group (n = 162). The researchers utilized multiple logistic regression and smoothed curve fitting to examine the relationship between urinary caffeine and caffeine metabolites and the incidence of stroke. The study found that higher urinary caffeine levels were associated with a lower risk of stroke in Mexican American participants (odds ratio [OR] = 0.886, 95% confidence interval [CI]: (0.791, 0.993), *P* = 0.037). After adjusting for certain participant characteristics, it was also found that higher urinary paraxanthine levels were associated with a lower risk of stroke incidence (OR = 0.991, 95% CI (0.984, 0.999), *P* = 0.027). Meanwhile, the highest urinary paraxanthine levels group had 43.7% fewer strokes than the lowest level group (OR = 0.563, 95% CI (0.341, 0.929), *P* = 0.025). In this study, we showed a negative link between urine paraxanthine levels and the risk of stroke. Meanwhile, urinary caffeine levels were negatively associated with the incidence of stroke in Mexican Americans, but no correlation in other populations. Our findings may have predictive and diagnostic implications in clinical practice. Further extensive prospective investigations are still needed to validate our conclusions.

## Introduction

Stroke is an acute cerebrovascular disease that can cause limb paralysis, speech impairment, impaired consciousness, and death. The burden of stroke is increasing^1,2^, and in 2019 it will continue to be the third greatest cause of death and disability combined (5.7% [5.1–6.2] of total disability-adjusted life years) and the second major cause of death (11.6% [10.8–12.2] of total deaths)^3^. Therefore, it is crucial from a therapeutic perspective to recognize and treat stroke patients' risk factors. Old age, diabetes, high cholesterol, smoking, a high body mass index (BMI), coronary heart disease, high blood pressure, etc.^3–6^.

Coffee beans, tea leaves, cocoa beans, and cola nuts are among the plants that contain caffeine (chemical name: 1,3,7-trimethylxanthine), an alkaloid. We consume caffeine daily, mainly through food and drinks (e.g., chocolate, coffee, tea, and cola drinks)^7,8^. Studies have shown that coffee is currently consumed by many age groups in the U.S. population and that daily caffeine intake is relatively stable over the week, with no significant differences between weekdays and weekend days^9^. Several diseases have been reported to be associated with caffeine consumption, including bone density issues, high blood pressure, cardiovascular disease, reproductive and developmental abnormalities, mental and behavioral disorders, and various cancers^10,11^. Caffeine is a central nervous system stimulant and is believed to provide health benefits in cardiovascular, cerebrovascular, and neurological diseases^12,13^. Some studies have reported that caffeine may be associated with antioxidant activity, regulation of metabolism, and improvement of endothelial function. More than three cups of coffee a day, according to a study, may lower your risk of developing Alzheimer's and Parkinson's disease^14^. Several studies have shown that caffeine consumption is associated with a reduced incidence of stroke^15^. Caffeine's antioxidant properties, which control cell proliferation and death, may be to blame for this. However, it is thought that caffeine consumption decreases cerebral blood flow in people who have had ischemic strokes, which has a negative therapeutic impact^13,16^.

Most studies have only focused on the effect of coffee consumption on stroke, and a few have evaluated the relationship between caffeine metabolites and stroke^17^. Studies on coffee consumption usually estimate caffeine content based on the diet type and volume/weight, while ignoring differences owing to raw material variety, origin, and production process; thus, caffeine consumption is less accurately recorded. Additionally, self-reported caffeine intake, which is prone to significant error, has hampered earlier research on the long-term effects of caffeine^18^. A trustworthy indicator of caffeine use is the amount of excreted caffeine and caffeine metabolites in urine, and information about the physiological endpoints of the metabolites clarifies the evidence linking the original chemical and the underlying condition^18^. This may lead to some bias in determining the relationship between caffeine and stroke. Additionally, individual variations in caffeine metabolism were overlooked, which may have led to varying concentrations of circulating caffeine and caffeine metabolites^19^. To further understand the connection between urine caffeine and caffeine metabolites and the likelihood of stroke, we conducted a cross-sectional study utilizing information from the National Health and Nutrition Examination Survey (NHANES) (2009–2014).

## Materials and methods

### Study population

NHANES is a cross-sectional nutrition survey in the USA that uses a multistage stratified probability sampling method^20^. The NHANES has received approval from the National Center for Health Statistics to collect and disseminate data. Adults (aged ≥ 20 years) who participated in NHANES from 2009 to 2014 were selected for this study. Individuals without prior information on urinary caffeine and caffeine metabolites and stroke. Authors cannot access information that could identify individual participants during or after data collection. The detailed NHANES study design and data are publicly available at https://www.cdc.gov/nchs/nhanes/.

**Figure 1:**
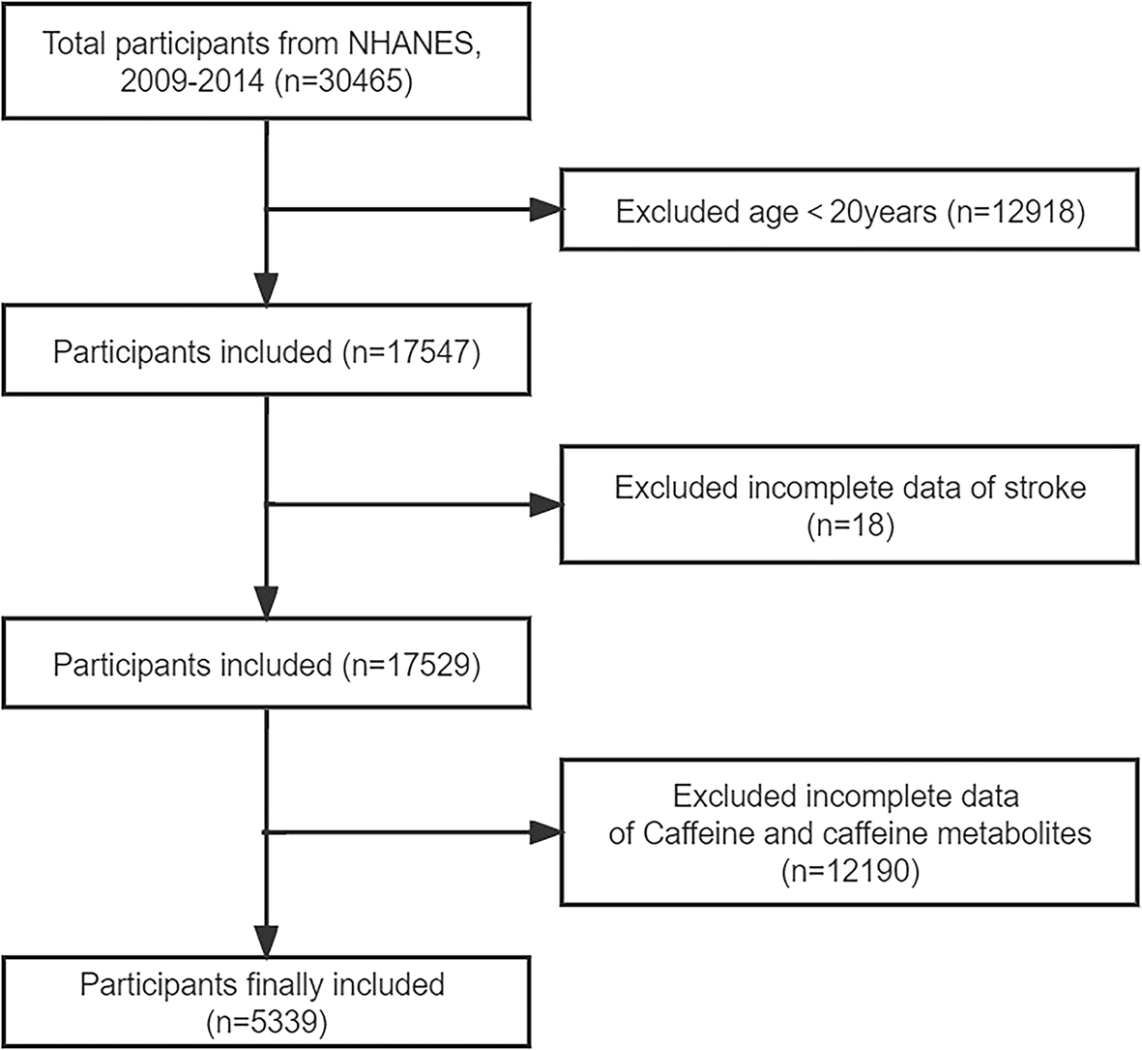
Flowchart of participants selection.

Data from a total of 30,465 participants between 2009 and 2014 were initially included in this study. Participants younger than 20 years old were excluded (n = 12,918). Participants with incomplete data of stroke (n = 18), and caffeine and caffeine metabolites (n = 12,190) were excluded. Finally, 5339 participants were included (Fig. 1).

### Exposure variable

In this study, urinary levels of caffeine and caffeine metabolites were used as the exposure variable. Theobromine (3,7-dimethylxanthine), theophylline (1,3-dimethylxanthine), and paraxanthine (1,7-dimethylxanthine) are the primary caffeine metabolites in humans^21^. After a 9-h overnight fast, urine samples for the study participants were taken during the morning session^22–24^. The National Center for Environmental Health, Division of Laboratory Sciences, Centers for Disease Control and Prevention, Atlanta, GA received the processed, stored, and sent urine samples for examination^23^. Using high-performance liquid chromatography-electrospray ionization-tandem quadrupole mass spectrometry (HPLC–ESI–MS/MS) and internal standards labeled with stable isotopes, caffeine and its metabolites are measured in urine^23^. The data were reviewed. The performing laboratory received uncertain values or incomplete data for verification^23^.

### Outcome variable

Using the Computer-Assisted Personal Interviewing (CAPI) technology, stroke history was gathered from patients by trained interviewers. The survey questionnaire is available on the NHANES website. Consistency checks are included into the CAPI system to minimize data entry errors. The information was checked and updated for consistency, completeness, and unreasonable values.

### Covariates

This study included covariates that may influence the relationship between caffeine and caffeine metabolites and stroke, including data on sex, age, race, history of high blood pressure, history of high cholesterol levels, history of diabetes, history of coronary heart disease, smoking at least 100 cigarettes in life (%), having 4/5 or more drinks every day, and BMI (kg/m^2^). BMI is calculated by weight/height squared. The Mobile Examination Center's qualified health technicians collected the body measurements data. Other covariates were collected through the CAPI system as the outcome variables. Details about covariates are available on the NHANES website (http://www.cdc.gov/nchs/nhanes/).

### Statistical analysis

With a *P*-value < 0.05 being considered significant, we ran the analyses using the software R (http:// www.Rproject.org) and EmpowerStats (http:// www. empowerstats.com). Missing continuous variables are excluded, while missing categorical variables form a separate classification. Measurement data were described as mean ± standard deviation (mean ± SD), and comparisons between groups were made using independent sample t-tests. Data that did not conform to a normal distribution differences between groups were compared using the Kruskal–Wallis rank sum test. An enumeration dataset was described as n (%), and chi-square tests were conducted to compare differences between groups. Multivariate logistic regression analysis was performed to evaluate the association between urinary levels of caffeine and caffeine metabolites and stroke. An unadjusted model (Model 1) was then created, followed by an adjusted model (Model 2) using variables including sex, age, and race or ethnicity. We obtained Model 3 by adjusting for sex, age, race, history of high blood pressure, history of high cholesterol levels, history of diabetes, history of coronary heart disease, smoking at least 100 cigarettes in life (%), having 4/5 or more drinks every day, and BMI (kg/m^2^). The relationship between caffeine and its metabolites in urine and stroke was further clarified by analysing various covariates. A simultaneous smoothed curve fitting was done to further investigate the association between caffeine and caffeine metabolite levels and stroke after controlling for a number of variables.

### Ethical approval

The data for this study were obtained from NHANES website. Therefore, it does not need to be approved by the Institutional Review Board (or Ethics Committee).

## Results

### The characteristics of participants

The final participants included were divided into a control group (n = 5135) and a stroke group (n = 162). Significant differences in baseline characteristics were observed between the two groups, except for sex, BMI, and urinary caffeine. The participants in the stroke group were older (64.080 ± 14.251 vs. 48.461 ± 17.286 years, *P* < 0.001) and had lower caffeine metabolites levels in urine (all *P* < 0.001) than those in the control group. Participants in the stroke group were more likely to have high blood pressure, high cholesterol levels, diabetes, coronary heart disease, and a history of smoking and drinking (all *P* < 0.001). In addition, there are some differences in the ethnic distribution of the two groups (*P* = 0.002) (Table 1).

**Table 1:** Basic characteristics and urinary levels of caffeine and caffeine metabolites in study participants.

Variables	Control group (n = 5135)	Stroke group (n = 162)	*P*-value
Age (years)	48.461 ± 17.286	64.080 ± 14.251	< 0.001
Sex			0.510
Man	2496 (48.608%)	83 (51.235%)	
Woman	2639 (51.392%)	79 (48.765%)	
Race			0.002
Mexican American	727 (14.158%)	11 (6.790%)	
Other Hispanic	516 (10.049%)	12 (7.407%)	
Non-Hispanic White	2197 (42.785%)	83 (51.235%)	
Non-Hispanic Black	1074 (20.915%)	45 (27.778%)	
Other Race	621 (12.093%)	11 (6.790%)	
High blood pressure			< 0.001
Yes	1776 (34.586%)	123 (75.926%)	
No	3350 (65.239%)	39 (24.074%)	
Unknown	9 (0.175%)	0 (0.00%)	
High cholesterol level			< 0.001
Yes	1616 (31.470%)	94 (58.025%)	
No	2935 (57.157%)	59 (36.420%)	
Unknown	584 (11.373%)	9 (5.556%)	
Diabetes			< 0.001
Yes	562 (10.944%)	46 (28.395%)	
No	4446 (86.582%)	112 (69.136%)	
Borderline	122 (2.376%)	4 (2.469%)	
Unknown	5 (0.097%)	0 (0.000%)	
Coronary heart disease			< 0.001
Yes	192 (3.739%)	29 (17.901%)	
No	4930 (96.008%)	131 (80.864%)	
Unknown	13 (0.253%)	2 (1.235%)	
Smoking			< 0.001
Yes	2211 (43.057%)	103 (63.580%)	
No	2923 (56.923%)	59 (36.420%)	
Unknown	1 (0.019%)	0 (0.000%)	
Drinking			< 0.001
Yes	694(13.515%)	39 (24.074%)	
No	3305 (64.362%)	89 (54.938%)	
Unknown	1136 (22.123%)	34 (20.988%)	
BMI (kg/m2 )	29.092 ± 6.921	29.625 ± 7.781	0.535
Caffeine (μmol/L)	7.689 ± 10.307	8.200 ± 10.920	0.802
Theophylline (μmol/L)	2.995 ± 10.124	2.596 ± 6.945	0.001
Paraxanthine (μmol/L)	26.612 ± 29.874	19.088 ± 21.566	< 0.001
Theobromine (μmol/L)	28.384 ± 40.184	21.692 ± 28.559	0.010

Mean ± SD for continuous variables: *P* value was calculated by logistic regression model.

% for categorical variables: *P* value was calculated by chi-square test.

BMI, body mass index.

### The association between urinary caffeine levels and stroke.

**Figure 2:**
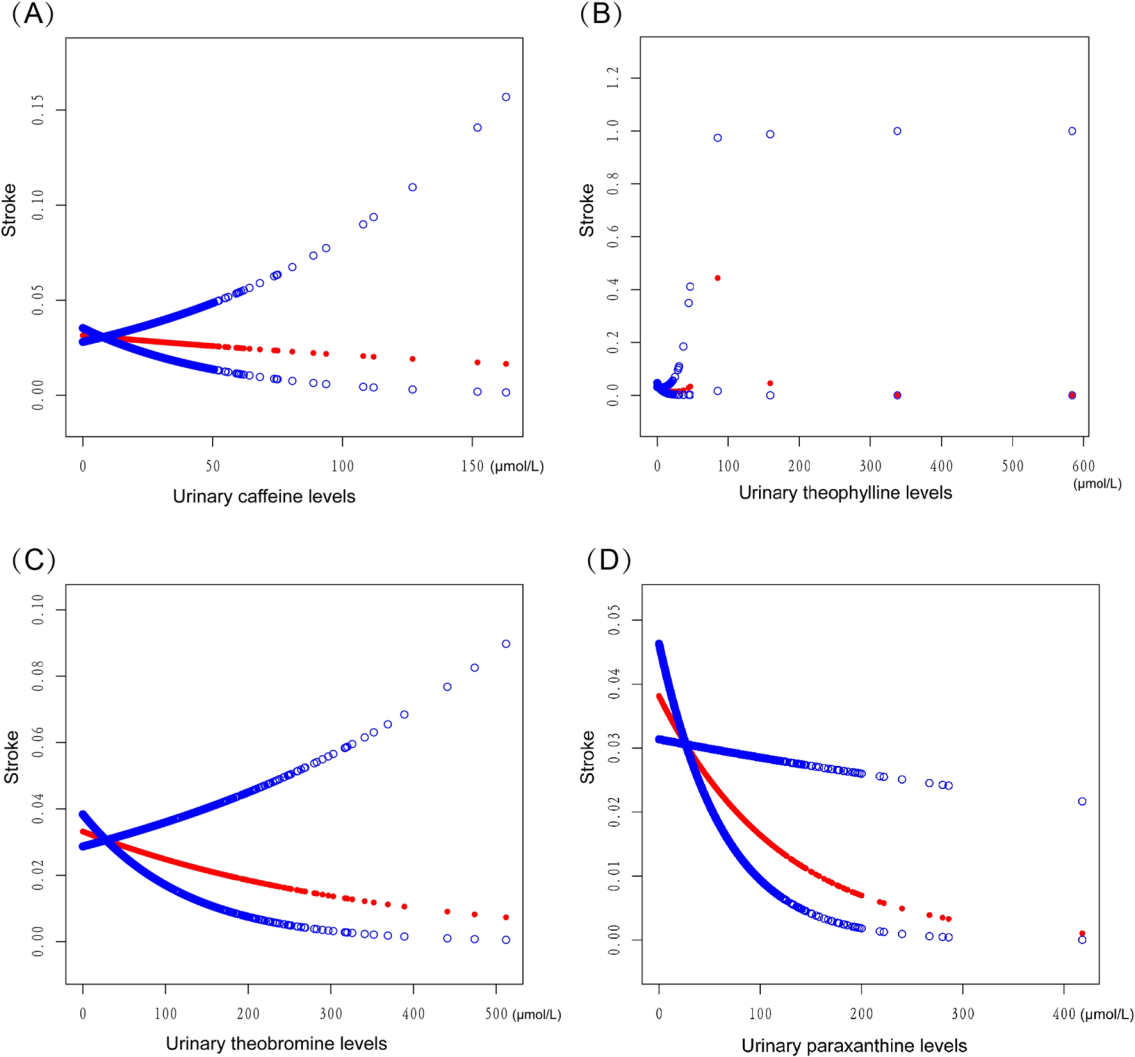
Smooth curve fittings of the association between urinary caffeine and caffeine metabolite levels (μmol/L) and stroke incidence. The red line represents the smoothed curve fitting between caffeine and caffeine metabolite and stroke. The blue line represents its 95% confidence interval. Sex, age, and race, high blood pressure, high cholesterol level, diabetes, coronary heart disease, smoking, drinking, BMI of participants were adjusted.

The results of the relationship between urinary caffeine levels and stroke are displayed in Table 2. No adjustment and adjustment for different covariates, urinary caffeine levels were consistently not statistically significant with stroke (all *P* > 0.05). The same results were then obtained by transforming continuous caffeine levels into categorical variables(quartiles) for the four subgroups (all *P* > 0.05) . Then, stratified analysis by age, sex and race was performed. In racial subgroup analysis, urinary caffeine levels were negatively associated with stroke occurrence in Mexican American (odds ratio [OR] = 0.886, 95% confidence interval [CI]: (0.791, 0.993), *P* = 0.037). When urinary caffeine levels were reduced by one unit(μmol/L), the incidence of stroke was reduced by 11.4%. The findings in other subgroups revealed no conclusive link between urine caffeine metabolites and stroke (all *P* > 0.05). Figure 2A displays the smooth curve fittings of the relationship between urine caffeine levels and the incidence of stroke.

**Table 2:** The association between urinary caffeine levels (μmol/L) and stroke.

	Model 1 *OR* (95% *CI*) *P* value	Model 2 *OR* (95% *CI*) *P* value	Model 3 *OR* (95% *CI*) *P* value
Caffeine (μmol/L)	1.004 (0.991, 1.018) 0.534	0.999 (0.984, 1.013) 0.847	0.996 (0.981, 1.011) 0.606
Caffeine categories			
Q1	Reference	Reference	Reference
Q2	0.799 (0.515, 1.240) 0.316	0.780 (0.496, 1.224) 0.280	0.758 (0.478, 1.202) 0.239
Q3	0.757 (0.485, 1.183) 0.222	0.713 (0.450, 1.129) 0.149	0.645 (0.402, 1.035) 0.069
Q4	0.948 (0.622, 1.443) 0.802	0.816 (0.526, 1.265) 0.363	0.747 (0.475, 1.175) 0.207
Age categories			
20–34	1.000 (0.900, 1.112) 0.996	0.993 (0.892, 1.105) 0.894	0.998 (0.883, 1.128) 0.973
35–49	0.991 (0.943, 1.042) 0.722	0.993 (0.943, 1.045) 0.782	0.994 (0.946, 1.044) 0.808
50–64	0.990 (0.962, 1.019) 0.495	0.989 (0.960, 1.019) 0.468	0.984 (0.953, 1.016) 0.311
65–80	1.000 (0.982, 1.017) 0.965	1.001 (0.983, 1.019) 0.941	1.001 (0.982, 1.020) 0.950
Sex			
Men	1.000 (0.977, 1.022) 0.970	0.990 (0.966, 1.014) 0.417	0.985 (0.961, 1.010) 0.244
Women	1.008 (0.991, 1.025) 0.365	1.003 (0.986, 1.021) 0.717	1.002 (0.983, 1.022) 0.838
Race			
Mexican American	0.971 (0.889, 1.060) 0.509	0.952 (0.872, 1.038) 0.264	0.886 (0.791, 0.993) 0.037
Other Hispanic	1.022 (0.971, 1.076) 0.396	1.007 (0.951, 1.065) 0.820	0.998 (0.935, 1.066) 0.959
Non-Hispanic White	1.010 (0.994, 1.025) 0.210	1.006 (0.991, 1.022) 0.429	1.005 (0.988, 1.022) 0.594
Non-Hispanic Black	0.955 (0.899, 1.014) 0.132	0.941 (0.885, 1.001) 0.054	0.944 (0.887, 1.004) 0.065
Other Race	1.017 (0.960, 1.078) 0.565	1.020 (0.956, 1.088) 0.556	1.022 (0.949, 1.099) 0.569

**Model 1**: The covariates were not adjusted.

**Model 2**: The age, sex, and race of the participants were adjusted.

**Model 3**: The age, sex, race, high blood pressure, high cholesterol level, diabetes, coronary heart disease, smoking, drinking, BMI of participants were adjusted.

### The association between urinary caffeine metabolites levels and stroke.

The results of the relationship between urinary caffeine metabolites levels and stroke are displayed in Table 3. No adjustment and adjustment for different covariates, urinary theophylline and theobromine levels were consistently not statistically significant with stroke (all P > 0.05). Then, conversion of continuous urinary theophylline and theobromine levels into categorical variables (quartiles). In Model 1, stroke incidence was reduced in the highest urinary theophylline (OR = 0.574, 95% CI (0.363, 0.907), *P* = 0.017) and theobromine (OR = 0.609, 95% CI (0.386, 0.960), *P* = 0.033) group compared to the lowest group. After adjusting for covariates, urinary theophylline and theobromine levels were not statistically significant with stroke incidence. Smooth curve fittings of the association between urinary theophylline and theobromine levels and stroke incidence are shown in Fig. 2B,C.

**Table 3:** The association between urinary caffeine metabolites levels (μmol/L) and stroke.

	Model 1 *OR* (95% *CI*) *P* value	Model 2 *OR* (95% *CI*) *P* value	Model 3 *OR* (95% *CI*) *P* value
Theophylline (μmol/L)	0.981 (0.931, 1.033) 0.465	0.991 (0.955, 1.028) 0.626	0.994 (0.958, 1.032) 0.767
Theophylline categories			
Q1	Reference	Reference	Reference
Q2	0.889 (0.593, 1.335) 0.571	0.900 (0.592, 1.367) 0.620	0.886 (0.578, 1.357) 0.577
Q3	0.672 (0.434, 1.041) 0.075	0.704 (0.449, 1.105) 0.127	0.678 (0.427, 1.075) 0.099
Q4	0.574 (0.363, 0.907) 0.017	0.635 (0.395, 1.021) 0.061	0.632 (0.388, 1.029) 0.065
Paraxanthine (μmol/L)	0.988 (0.981, 0.995) 0.001	0.991 (0.984, 0.998) 0.017	0.991 (0.984, 0.999) 0.027
Paraxanthine categories			
Q1	Reference	Reference	Reference
Q2	0.870 (0.584, 1.269) 0.492	0.844 (0.559, 1.273) 0.419	0.855 (0.562, 1.301) 0.239
Q3	0.631 (0.410, 0.973) 0.037	0.672 (0.430, 1.048) 0.080	0.658 (0.417, 1.038) 0.069
Q4	0.470 (0.292, 0.755) 0.002	0.549 (0.336, 0.895) 0.016	0.563 (0.341, 0.929) 0.025
Theobromine (μmol/L)	0.994 (0.998,1.000) 0.035	0.996 (0.990, 1.001) 0.131	0.997 (0.992, 1.002) 0.270
Theobromine categories			
Q1	Reference	Reference	Reference
Q2	0.874 (0.578, 1.320) 0.522	0.943 (0.617, 1.440) 0.785	0.928 (0.602, 1.429) 0.733
Q3	0.729 (0.473, 1.123) 0.152	0.826 (0.528, 1.294) 0.405	0.836 (0.529, 1.320) 0.441
Q4	0.609 (0.386, 0.960) 0.033	0.725 (0.452, 1.163) 0.183	0.811 (0.501, 1.313) 0.393

**Model 1:** The covariates were not adjusted.

**Model 2:** The age, sex, and race of the participants were adjusted.

**Model 3:** The age, sex, race, high blood pressure, high cholesterol level, diabetes, coronary heart disease, smoking, drinking, BMI of participants were adjusted.

Model 1 showed that increased levels of urinary paraxanthine levels were associated with a decreased incidence of stroke (OR = 0.988, 95% CI (0.981, 0.995), *P* = 0.001). Model 2 also showed a significant correlation between urinary paraxanthine levels and stroke incidence after adjusting for covariates (OR = 0.991, 95% CI (0.984, 0.998), *P* = 0.017). In Model 3, when age, sex, race, high blood pressure, high cholesterol level, diabetes, coronary heart disease, smoking, drinking, BMI of participants were adjusted, the same significant correlation was shown (OR = 0.991, 95% CI (0.984, 0.999), *P* = 0.027). For multiple regression analysis, we further transformed urinary paraxanthine levels from a continuous variable to a categorical variable (four categories). In the second highest urinary paraxanthine levels group(Q2), urinary paraxanthine levels was not statistically significant with stroke incidence, with or without adjustment for covariates. In the third highest urinary paraxanthine levels group(Q3), urinary paraxanthine levels were negatively associated with a reduction in stroke incidence in Model 1(OR = 0.631, 95% CI (0.410, 0.973), *P* = 0.037), but not statistically significant in the other models(all *P* > 0.05). In the unadjusted covariate models, the highest urinary paraxanthine levels group(Q4) was associated with a reduced incidence of stroke compared with the lowest levels group (OR = 0.470, 95% CI (0.292, 0.755), *P* = 0.002). When adjusting for different covariates, this correlation was still present in Models 2(OR = 0.549, 95% CI (0.336, 0.895), *P* = 0.016) and Models 3(OR = 0.563, 95% CI (0.341, 0.929), *P* = 0.025). After adjusting for a number of covariates, the highesturinary paraxanthine levels group(Q4) had 43.7% fewer strokes than the lowest level group. Figure 2D displays the smooth curve fittings of the relationship between urine paraxanthine levels and the incidence of stroke.

## Discussion

After analyzing the data from a large sample from a 6-year cross-sectional study, we observed that urinary caffeine levels were negatively associated with stroke occurrence in Mexican American, but no correlation in other populations. In addition, urinary paraxanthine levels were negatively associated with the incidence of stroke. Few prior long-term population-based investigations, to our knowledge, have looked at the relationship between standardized measurements of urine caffeine and caffeine metabolites with stroke. Our findings not only provide objective evidence for clinical and basic research, but also have implications for the identification and intervention of stroke risk factors.

Previous studies on NHANES data explored the relationship between stroke and caffeine consumption at the coffee consumption level^25^. They concluded that an increase in daily coffee consumption was associated with a decrease in stroke incidence. And some studies have shown that coffee consumption is safe and does not increase the risk of death^15^. We considered the diversity of sources of caffeine, with some variation in the types of caffeine-containing foods, ingredients and preparation processes. And self-reported caffeine consumption is not sufficiently accurate, which leaves a large margin of error in the estimation of caffeine content, which is difficult to estimate^18,26^. Caffeine has a half-life of 2.5–4.5 h in humans^27,28^, so after 9 h of fasting, the difference in caffeine levels in the samples will be further reduced, and therefore the effect of differences in the timing and frequency and amount of final caffeine consumption on the levels of caffeine and its metabolites in urine will be further reduced. Therefore, our study starts from a relatively stable urine sample to clarify the relationship between caffeine and its metabolites and stroke by analysing the levels of caffeine and its metabolites in urine at fixed time points. Our findings are consistent with those of previous studies and further demonstrate the reliability of our research methods. However, a multicenter case-crossover study showed that the relative risk (RR) of stroke within 1 h of coffee consumption was 2.0 (RR = 2.0, 95% CI (1.4–2.8), *P* < 0.001), which differed from our findings^29^. This difference can be attributed to several factors. First, the study compared each participant’s coffee consumption in the hour before stroke symptoms with his or her usual frequency of consumption in the previous year, which differed somewhat from the metabolic cycle of caffeine. Second, coffee consumption is not a good proxy for caffeine intake because of the different coffee prices and types. Self-reported caffeine intake has been found to be weakly correlated with measured caffeine levels, according to several studies^18,26^, which can be attributed to the influence of the numerous components listed above.

We showed in this extensive cross-sectional investigation that urine caffeine levels were negatively correlated with the incidence of stroke among Mexican Americans, while there was no association in other populations. This may be due to differences in the incidence of stroke by race^30,31^. In addition, there are some differences in the dietary habits of different races, where food intake is accompanied by not only caffeine but also other substances, which may influence the occurrence of a stroke. Previous studies have found that CYP1A2 is responsible for over 90% of caffeine me-tabolism^32^. Individuals with genotype AA are extensive metabolizers of caffeine, those with genotype AC are in-termediate metabolizers of caffeine, and those with genotype CC are low metabolizers of caffeine^33^. Several studies have shown that CYP1A2 variants differ in different ethnic groups^34,35^. However, there are no relevant studies on CYP1A2 variants in Mexican-Americans. We hope that based on our findings, more scholars will conduct more in-depth studies on the polymorphisms of CYP1A2 in different ethnic groups in the future. A study of a Chinese population, showed that Individuals with CYP1A2 rs762551 C was associated with a lower risk of stroke than that of allele A^36^. In individuals with the CYP1A2 rs762551 AC + CC genotype, coffee consumption was associated with lower odds of hypertension^37^. Since hypertension is CYP1A2 variants risk factor for stroke, the study suggests that CYP1A2 variants may be indirectly associated with the development of stroke. The specific mechanisms that link CYP1A2 variants to stroke occurrence still require further in-depth study in the future. Several studies showing that sex-specific activity and expression of the P450 1A2 (CYP1A2) gene were related to caffeine metabolism^16,38–40^. A study of ischemic stroke involving 270 (55%) male and 229 (45%) female participants, no sex differences in the severity of the stroke, stroke subtype, or infarct size and location were reported. However, the results showed a higher mortality rate in female participants^41–43^. However, no sex-related differences were observed in our study for the time being. More high-quality randomised controlled studies with large sample sizes are needed in the future to investigate gender differences in stroke risk factors.

Additionally, the incidence of stroke had a negative relationship with urine paraxanthine levels. Some findings may explain our results. First, caffeine, which is an adenosine receptor antagonist, has been reported to exert neuroprotective effects. It attenuates dopaminergic toxicity and decreases inflammation by up regulating the A1 receptors^20^. Second, caffeine consumption was found to modulate stroke risk factors such as blood glucose and lipid levels^24,25,44,45^. This result indirectly proves that caffeine intake plays a crucial role in reducing stroke occurrence. Third, endothelial cell injury is an important cause of arterial thrombosis. Caffeine has antioxidant effects and can scavenge reactive oxygen species, especially -OH^46,47^. These effects could prevent endothelial cell damage, thus reducing the incidence of stroke. Fourth, habitual caffeine intake could increase sympathetic nerve activity, circulating catecholamines^48^, and arterial blood pressure^49,50^. This could lead to an increase in the cardiovascular reserve to maintain arterial blood pressure and enhance the tolerance of the central nervous system to the insufficient blood supply, which helps reduce the occurrence of ischemic stroke. In addition, foods containing caffeine may contain various other chemicals. For instance, tea polyphenols, catechin polymers, tea polysaccharides, and other components, such as chlorogenic acid and phenolic compounds, are found in coffee^51,52^. These compounds may have anti-inflammatory, anti-thrombotic, and anti-inflammatory properties in addition to controlling metabolism, reducing blood sugar and cholesterol levels, enhancing gut flora, increasing endothelial function, and promoting antioxidant activity^52,53^. Finally, a study showed that increased caffeine intake reduced DNA methylation levels, which were positively correlated with biological age, a stronger risk factor for stroke than age^54^. This finding further indicated that increased caffeine intake may lower biological age, thus reducing the occurrence of stroke. However, based on the above-mentioned speculations and findings, the exact relationship between caffeine and caffeine metabolites and stroke cannot be determined, and more relevant studies are needed.

Our research advantage lies mainly in the following several areas. First, it is a large sample analysis from NHANES data, which may minimize bias owing to insufficient sample size. Second, data were stratified by sex, age, and race and the sample in the current study was analyzed using multiple regression analysis after multiple confounders were taken into account, including age, sex, race, high blood pressure, high cholesterol, diabetes, coronary heart disease, smoking, drinking, and BMI of participants. We examined a number of risk factors that are frequently linked to stroke, which may offer more reliable proof of a connection between caffeine and caffeine metabolites in urine and the incidence of stroke Thirdly, to avoid recall bias, misclassification, and kind and preparation of food, our exposure variables were evaluated by urine levels of caffeine and caffeine metabolites rather than self-reported caffeine-containing food intake. There are a few restrictions on this study. Firstly, because this was a cross-sectional study, it is not possible to conclude that caffeine metabolites cause stroke based solely on the results shown above. Further longitudinal studies are required to address this question. Second, the participants in this study were mainly from the U.S., which is not representative of the global population. Thus, more multi-regional and multi-center studies are needed to further investigate the relationship between stroke incidence and caffeine metabolite levels. Third, as the NHANES database does not distinguish between types of stroke, our study can only suggest an association between caffeine and its metabolites and stroke, and does not clarify whether the stroke was haemorrhagic or ischaemic. Finally, due to the way the NHANES data were stratified and the inherently lower incidence of stroke, the number of participants in the stroke group was smaller than in the control group, which may have caused some bias in the study results. However, the development of different models and the analysis of multiple covariates have led to more reliable results on the relationship between caffeine and its metabolites and stroke. More accurate randomized controlled trials are required to validate the results of this study.

## Conclusions

In this study, we showed a negative link between urine paraxanthine levels and the risk of stroke. Meanwhile, urinary caffeine levels were negatively associated with the incidence of stroke in Mexican Americans, but no correlation in other populations. Our findings may have predictive and diagnostic implications in clinical practice. Further extensive prospective investigations are still needed to validate our conclusions.

## Data Availability

The original data were accessed from https://www.cdc.gov/nchs/nhanes/about_nhanes.
